# Screening for heterogeneous drug resistance in tuberculosis and its impact on clinical prognosis: A comprehensive review

**DOI:** 10.1016/j.isci.2026.115049

**Published:** 2026-02-17

**Authors:** Xiaoyun Du, Keqing Shi, Hao Zhang, Yingzhi Chong

**Affiliations:** 1 Weifang People’s Hospital, Shandong Second Medical University, Weifang 261053, Shandong, China; 2 School of Public Health, Shandong Second Medical University, Weifang 261053, Shandong, China

**Keywords:** health sciences, immunology, medical microbiology, medical specialty, medicine

## Abstract

Tuberculosis (TB) remains a leading global health threat, with the rise of drug-resistant (DR-TB) strains posing a significant impediment to disease control. An increasingly recognized and complex challenge is heteroresistance, the coexistence of drug-susceptible and drug-resistant *Mycobacterium tuberculosis* subpopulations within a single host. This phenomenon acts as a crucial intermediate in the evolution toward fixed resistance and has been strongly associated with poor clinical prognoses, including treatment failure and the amplification of resistance. This review synthesizes the current state of knowledge regarding the screening methodologies for and the adverse outcomes associated with TB heteroresistance. The diagnostic gap creates a substantial risk of misclassifying patients and prescribing functionally inadequate therapeutic regimens. Further, the presence of heteroresistance significantly correlates with diminished treatment efficacy and an increased likelihood of unfavorable outcomes. Importantly, the precise clinical significance of low-frequency resistant variants remains a critical area of investigation, with an urgent need to establish evidence-based thresholds to guide therapy. Future research must focus on defining clinically relevant heteroresistance thresholds, standardizing advanced diagnostic platforms, and further elucidating the biological mechanisms that govern the emergence and persistence of these heteroresistance bacterial populations to ultimately improve patient outcomes and curb the spread of drug-resistant tuberculosis.

## Graphical abstract

**Figure:**
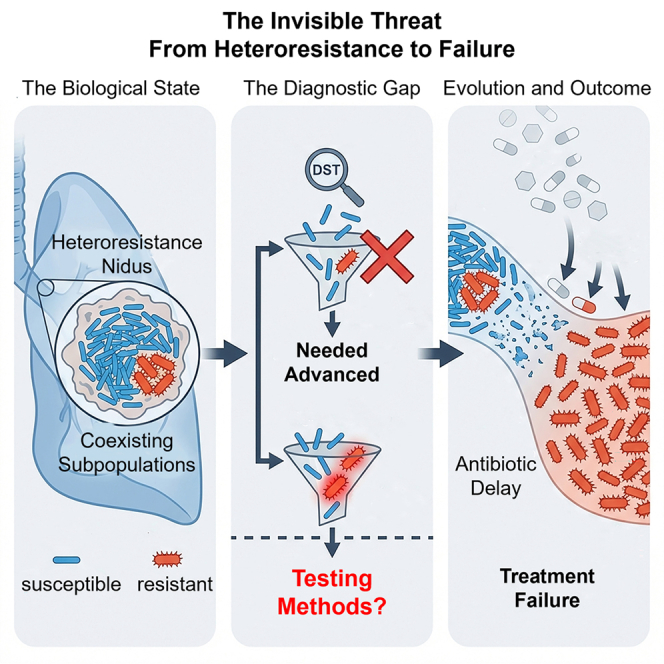

## Introduction

Despite decades of concerted global efforts, tuberculosis (TB), caused by *Mycobacterium tuberculosis* (MTB), remains a formidable public health crisis and a leading cause of death from a single infectious agent worldwide. The challenge of controlling TB has been profoundly exacerbated by the emergence and spread of drug-resistant strains, particularly multidrug-resistant TB (MDR-TB), which is resistant to the two most potent first-line anti-TB drugs, isoniazid, and rifampicin.^1^ The global response to DR-TB is hampered by diagnostic delays, arduous and toxic treatment regimens, and suboptimal cure rates, posing a significant threat to achieving the goals of the end TB strategy.^2^ In recent years, a more insidious and complex phenomenon known as heteroresistance has gained increasing recognition as a critical, yet often overlooked, driver of treatment failure and the evolution of widespread drug resistance.^3^^,^^4^

Heteroresistance is defined as the coexistence of both drug-susceptible and drug-resistant MTB subpopulations within a single infected individual.^2^^,^^4^ This state represents a crucial evolutionary stepping stone, a gray zone between full susceptibility and fixed, high-level resistance. The clinical implications of this phenomenon are substantial; the presence of even a minor resistant subpopulation, which may be undetectable by conventional diagnostic methods, can be selected for under the pressure of standard antibiotic therapy, leading to the amplification of the resistant clone and subsequent treatment failure or relapse.^3^^,^^5^ Several studies have highlighted this perilous link; for example, investigations by Kargarpour Kamakoli et al. and Shin et al. have demonstrated a significant association between mixed-strain infections or heteroresistance and adverse treatment outcomes.^5^^,^^6^ This challenge is compounded by the diagnostic conundrum it presents. Standard phenotypic drug susceptibility testing (DST), historically considered the gold standard, typically defines resistance at a threshold of 1% of the bacterial population, but its long turnaround time is a major limitation.^7^ Conversely, while rapid molecular diagnostics have revolutionized TB testing, many established platforms, including Sanger sequencing and even some real-time PCR-based assays, exhibit insufficient analytical sensitivity to reliably detect resistant subpopulations that fall below a 5%–20% frequency, creating a dangerous diagnostic blind spot.^8^^,^^9^^,^^10^ This discrepancy between genotypic and phenotypic results, often attributed to the presence of low-frequency resistant variants, complicates clinical decision-making and underscores the urgent need for more sensitive diagnostic tools.^11^

The growing body of evidence implicating heteroresistance in poor clinical prognoses necessitates a comprehensive synthesis of the current state of knowledge. Therefore, the systematic literature search across PubMed/MEDLINE, and Web of Science databases from January 2010 to June 2025 were conducted. Search terms included combinations of: (“tuberculosis” or “MTB”’) and (“heteroresistance” or “mixed infection” or “heterogeneity”) and (“drug resistance” or “antimicrobial resistance”). Inclusion criteria were: (1) original research articles or systematic reviews; (2) studies reporting on TB heteroresistance detection, prevalence, or clinical outcomes; (3) publications in English or with English abstracts. Exclusion criteria included: (1) case reports with fewer than 5 patients; (2) studies focusing solely on non-tuberculous mycobacteria; (3) conference abstracts without full-text availability. Two reviewers independently screened titles and abstracts, with discrepancies resolved through discussion. Specifically, this paper will: (1) delineate the definition and prevalence of TB heteroresistance and discuss the challenges it poses to diagnosis and treatment; (2) critically evaluate and compare the spectrum of screening methods, from traditional phenotypic assays to cutting-edge genomic technologies like next-generation sequencing and digital PCR; (3) analyze the clinical impact of heteroresistance on treatment efficacy, failure, and recurrence, thereby clarifying its role in patient prognosis; and (4) discuss potential preventive and control measures. By consolidating current research, this review seeks to highlight critical knowledge gaps and propose future research directions, ultimately contributing to the development of improved strategies for the diagnosis and management of this complex form of drug-resistant tuberculosis.

## The landscape of TB heteroresistance: Prevalence and challenges

The phenomenon of heteroresistance introduces a layer of complexity to the biology and clinical management of tuberculosis that challenges conventional paradigms of drug resistance. Its understanding requires a nuanced appreciation of its definition, its epidemiological footprint, and the profound difficulties it poses for both diagnosis and therapy.

### Defining the concept: Heteroresistance vs. mixed infection

At its core, heteroresistance refers to the presence of a phenotypically or genotypically mixed population of *MTB* within a single patient, where subpopulations exhibit differential susceptibility to one or more antimicrobial agents.^3^^,^^4^ It is crucial to distinguish this from polyclonal or mixed-strain infection, where a patient is co-infected with two or more genetically distinct MTB strains that may have different susceptibility profiles.^5^^,^^12^ Heteroresistance, in its strictest sense, often describes the emergence of resistant mutants from a clonal, initially susceptible population during the course of infection and treatment—a process of within-host evolution.^3^^,^^13^ Zhang et al. provided a useful framework by analyzing FQ-resistant isolates, classifying heteroresistant populations into two groups: those comprising a mix of wild-type and mutant subpopulations, representing an early stage of resistance evolution, and those comprising multiple different resistant subpopulations, indicative of further evolution under sustained drug pressure.^14^ The source of heteroresistance can be primary, where a patient is infected with a mixed population from another individual, or acquired, where resistance emerges *de novo* within the host, often as a direct consequence of inadequate or suboptimal therapy.^3^^,^^15^

However, only phenotypic methods cannot definitively distinguish between heteroresistance and mixed infection. Previous studies employ different approaches to distinguish heteroresistance from mixed infection. Heteroresistance typically refers to resistance-conferring mutations arising within a clonal population, detectable as multiple alleles at the same genomic position (e.g., both wild-type and mutant sequences at *rpoB* codon 531). This is confirmed through: (1) single nucleotide polymorphism (SNP) analysis showing <12 SNP differences between subpopulations; (2) presence of heterozygous resistance-associated positions in sequencing data; (3) quantitative PCR showing varying proportions of wild-type and mutant alleles at specific loci. In contrast, mixed infections involve genetically distinct strains (>100 SNP differences), identified through: (1) MIRU-VNTR or spoligotyping showing multiple patterns; (2) whole genome sequencing (WGS) revealing distinct phylogenetic lineages.^9^^,^^16^^,^^17^^,^^18^

### Prevalence and global distribution

Determining the precise prevalence of heteroresistance is challenging, as reported rates are highly dependent on the diagnostic methodology employed, the specific drug being investigated, and the geographical region and patient cohort under study. A systematic review and meta-analysis conducted by Ye et al. provided a global estimate, reporting pooled prevalences of 7% for rifampicin (RIF), 5% for isoniazid (INH), and a notably higher 10% for fluoroquinolones (FQs).^4^ This finding, that FQ heteroresistance is more common, has been corroborated by multiple independent studies, highlighting the particular vulnerability of this critical class of second-line drugs.^19^^,^^20^

Regional studies reveal significant variations. For instance, investigations in Pakistan by Rashid et al. using WGS found a staggering 29% prevalence of bedaquiline heteroresistance, a concerning figure for a relatively new drug.^21^ Work from India has similarly documented notable rates; Desikan et al. reported an overall heteroresistance prevalence of 5.4% for RIF and INH in Central India using line probe assays (LPAs),^22^ while another study among tuberculous meningitis patients found heteroresistance rates of 4% for INH and 1% for RIF.^23^ In high-burden settings like South Africa, the rise of heteroresistance is alarming, with one study in the Eastern Cape Province observing an increase from 9.6% in 2018 to 32.2% in 2020.^24^ Due to the differences in research methods, the direct comparison of heterogeneous resistance rates in different studies is extremely complex. However, its relatively high rate of heterogeneous drug resistance is currently widely recognized.

### The dual challenge: Diagnosis and treatment

The existence of heteroresistance presents a formidable two-pronged challenge to TB control. The first is diagnostic: heteroresistant populations often exist below the limit of detection (LOD) of many rapid molecular tests. As Danchuk et al. compellingly demonstrated, the gold-standard phenotypic agar proportion method can detect resistant subpopulations at a 1% frequency, whereas the LOD for WGS and Xpert Ultra is around 10%, and for the classic Xpert, it can be as high as 60%.^10^ This analytical gap means a clinically significant resistant subpopulation can be easily missed, leading to a misclassification of a patient’s resistance status. This issue is a primary cause of discordant results between genotypic and phenotypic tests, a frequent predicament in clinical microbiology that complicates interpretative reporting.^11^

The second, and more consequential, challenge is therapeutic. When heteroresistance is undetected, patients are often prescribed a standard treatment regimen that is, in effect, functionally inadequate. This suboptimal therapy acts as a powerful selective pressure, suppressing the sensitive majority while allowing the pre-existing resistant minority to proliferate unimpeded.^3^ This dynamic can lead directly to treatment failure, disease relapse with a fully resistant strain, or the *in vivo* amplification of resistance.^13^ Furthermore, the long-term persistence of heteroresistance, which can last for months or even years as shown by Chen et al., challenges the notion that it is merely a transient state, suggesting that spatially segregated lung lesions may harbor and sustain distinct subpopulations, further complicating therapeutic eradication.^25^ Ultimately, heteroresistance serves as a biological mechanism that undermines treatment efficacy and fuels the broader epidemic of drug-resistant tuberculosis.

## Screening methods for TB heteroresistance

The accurate and timely detection of heteroresistance is the cornerstone of effective management, yet it remains a significant diagnostic challenge. The evolution of screening methodologies mirrors the growing understanding of this complex phenomenon, moving from traditional culture-based approaches to highly sensitive molecular techniques. This section provides a critical overview of the existing arsenal of screening methods, evaluating their principles, performance, and inherent limitations in identifying mixed MTB populations.

### Phenotypic screening methods

Phenotypic drug susceptibility testing (pDST), which directly assesses the ability of bacteria to grow in the presence of an antibiotic, has long been considered the “gold standard” for determining drug resistance. Its primary strength lies in its ability to detect resistance irrespective of the underlying genetic mechanism.

#### Culture-based drug susceptibility testing

Conventional pDST methods, such as the agar proportion method (APM) and liquid culture systems like the BACTEC mycobacteria growth indicator tube (MGIT) 960, are defined by their capacity to detect resistant subpopulations that constitute at least 1% of the total bacterial population.^10^ This analytical sensitivity makes them theoretically capable of identifying clinically relevant heteroresistance. Landmark studies by Folkvardsen et al. have systematically demonstrated that the MGIT system can reliably detect RIF and INH heteroresistance at frequencies of 1%–5% in experimentally mixed cultures, outperforming many molecular methods in sensitivity.^7^^,^^26^ The improved safety and rapidity of the modified microscopic observation drug susceptibility (MODS) assay, as reported by Fomda et al., also shows promise as a phenotypic tool with a turnaround time of just 9 days, making it suitable for resource-limited settings.^27^ However, the fundamental drawback of all culture-based methods is their protracted turnaround time, often taking several weeks to yield results, which is incompatible with the urgent need for rapid treatment decisions.^1^

The critical concentration used in phenotypic DST significantly influences heteroresistance detection. For rifampicin, the WHO-recommended critical concentration is 1.0 μg/mL for MGIT and 40 μg/mL for Löwenstein-Jensen medium.^28^^,^^29^ However, studies have shown that using lower concentrations (0.5 μg/mL for MGIT) can detect low-level resistance that may be clinically relevant.^30^ For isoniazid, the standard 0.1 μg/mL concentration may miss heteroresistance involving katG mutations that cf. low-level resistance.^31^ The revised critical concentrations for fluoroquinolones (moxifloxacin: 0.25 μg/mL; levofloxacin: 1.0 μg/mL) better capture heteroresistant populations compared to previous higher concentrations.^32^ These drug-specific considerations are crucial when interpreting discordant genotypic-phenotypic results, as heteroresistant populations near the critical concentration threshold may yield inconsistent phenotypic results.

#### Advanced bacteriological examination

Beyond standard DST, innovative methods focusing on the direct observation of bacterial growth dynamics have emerged. A prime example is the one-cell doubling evaluation of living arrays of mycobacterium (ODELAM) technique developed.^33^ This time-lapse microscopy-based method analyzes the growth of individual bacterial cells into microcolonies, allowing for the rapid, sequence-independent detection of phenotypic resistance. Remarkably, ODELAM was able to identify resistant colony-forming units present at a ratio as low as 1:1,000 within 48 h, successfully quantifying a clinically derived heteroresistant subpopulation that had been missed by WGS.^33^ This approach exemplifies a new frontier in phenotypic analysis, offering high sensitivity without the long delays of traditional culture.

### Genotypic screening methods

In response to the slowness of pDST, a variety of genotypic methods have been developed. These techniques detect specific genetic mutations known to cf. drug resistance, offering a significant advantage in speed, as many can be performed directly on clinical specimens.

#### PCR-based and hybridization techniques

The first wave of rapid molecular diagnostics included real-time PCR assays and LPAs. Commercial LPAs like GenoType MTBDRplus and MTBDRsl have been widely adopted for their ability to simultaneously detect MTB and mutations associated with resistance to first- and second-line drugs.^34^^,^^35^ Their ability to reveal “mixed patterns”—the simultaneous presence of both wild-type and mutant probe signals—provides a direct indication of heteroresistance.^34^^,^^36^ However, their analytical sensitivity is a critical limitation; studies have shown their LOD for heteroresistance is typically in the range of 5%–10%.^7^^,^^37^

Real-time PCR-based methods have evolved to enhance sensitivity. Techniques incorporating HRM analysis or “sloppy molecular beacons” have improved the detection of mixed populations, with some assays identifying mutant alleles down to a 5%–10% frequency.^38^^,^^39^ A significant advancement was reported with the “Deep Melt” assay, which uses a probe-clamping strategy to selectively enrich low-abundance mutant DNA, achieving a remarkable 1% detection limit for INH resistance-associated mutations.^40^ Despite these improvements, the widely deployed Xpert MTB/RIF and Xpert MTB/RIF Ultra assays, while revolutionary for TB diagnosis, have a comparatively high LOD for heteroresistance, ranging from 10% to as high as 70% depending on the specific mutation and assay version, creating a significant risk of missing low-frequency resistant subpopulations.^10^^,^^37^ The detection limits for heteroresistance vary significantly by specific rpoB mutation and Xpert version. For Xpert MTB/RIF, probe E (codons 529–533) has the poorest sensitivity for heteroresistance, requiring mutant frequencies >65% for reliable detection of S531L mutations. Probes A and B (covering codons 507–511 and 511–518) detect heteroresistance at 40%–50% frequency. Xpert Ultra shows improved performance: for H526Y mutations, the detection limit is ∼20% mutant frequency.^10^

#### Next-generation sequencing and droplet digital PCR

The advent of Next-generation sequencing (NGS) has fundamentally transformed the ability to characterize heteroresistance. Both untargeted WGS and targeted deep sequencing (TDS) can identify and, crucially, quantify resistant variants at very low frequencies. Studies utilizing these technologies have uncovered a much higher prevalence of heteroresistance than previously appreciated. For example, Operario et al. used amplicon-based NGS to find heteroresistance in 57% of MDR-TB isolates,^41^ while TDS revealed PZA heteroresistance at frequencies as low as 2%–7%, a level missed by both WGS and Sanger sequencing.^42^ The major hurdles for routine WGS, especially directly from sputum, are the high cost and the overwhelming presence of human DNA. This has spurred the development of enrichment strategies, such as the MycoCap DNA capture platform, which selectively isolates MTB DNA, and targeted amplicon sequencing approaches like Deeplex Myc-TB and Nanopore targeted sequencing, enabling comprehensive resistance profiling directly from clinical samples in a matter of days.^43^^,^^44^^,^^45^

Several WHO-endorsed targeted NGS platforms have emerged as scalable and clinically actionable solutions for comprehensive drug resistance profiling in MTB. Among these, the Deeplex Myc-TB assay (GenoScreen) employs multiplex PCR to amplify 18 gene targets associated with resistance to 13 anti-TB drugs, typically achieving >1000× coverage with sensitivity for variants present at ≥1%. It processes 24–96 samples per run within 24–48 h and has been successfully implemented in settings such as Moldova and Peru, where it detected heteroresistance of MDR-TB cases missed by conventional methods.^46^ Nanopore-based targeted sequencing utilizes portable MinION devices for real-time sequencing of PCR amplicons, offering variable coverage (100–5000×) depending on run duration and enabling results within 8–24 h for 1–12 samples per flow cell. This platform has been deployed in Uganda for bedaquiline resistance surveillance, demonstrating 92% concordance with Illumina sequencing in detecting heteroresistance.^47^ The ion AmpliSeq TB panel relies on semiconductor sequencing of multiplex PCR products, achieving >500 × average depth across targets for up to 32 samples per chip and reliably identifying heteroresistance at 3%–5% variant frequency in proficiency panels.^48^ Additionally, the MycoCap target enrichment approach uses hybridization capture to isolate MTB-specific sequences directly from complex samples such as sputum, even with <10% MTB DNA content, and has detected heteroresistance in 34% of culture-negative but Xpert-positive specimens.^49^ Collectively, these platforms bridge the critical gap between rapid but limited sensitivity of conventional molecular tests and the comprehensive yet slower turnaround of whole-genome sequencing, thereby enabling practical, routine detection of heteroresistance in clinical and public health settings.

For ultimate precision in quantification, ddPCR has emerged as an exceptionally powerful tool. By partitioning a sample into thousands of nanoliter-sized droplets, ddPCR enables the absolute quantification of wild-type and mutant DNA molecules. This allows for unparalleled sensitivity and accuracy in measuring the proportion of a resistant subpopulation. Pholwat et al. first demonstrated its potential by detecting resistant variants down to a 0.1% frequency.^50^ Subsequent work has refined this approach for various drugs, with Zheng et al. and Zhang et al. successfully developing ddPCR assays to detect and quantify INH heteroresistance with LODs well below the 1% clinical threshold.^51^^,^^52^

#### Technical considerations: Detection limits vs. variant-calling thresholds

It is essential to distinguish between analytical detection limits and variant-calling thresholds. The detection limit represents the lowest frequency at which a variant can be reliably detected above background noise, while the variant-calling threshold is the minimum frequency used to report a variant as present. For WGS at standard coverage (50–100×), the theoretical detection limit is ∼5%–10%, but most pipelines apply a conservative 10% calling threshold to minimize false positives.^53^ Deep sequencing (>1000× coverage) can detect variants at 0.1%–1% frequency, with calling thresholds typically set at 1%–3% depending on error rates.^54^ Digital PCR has a detection limit of 0.01%–0.1% determined by the number of partitions analyzed, with calling thresholds often set at 0.5% to ensure specificity.^55^^,^^56^ These thresholds directly impact reported prevalence rates: studies using a 1% threshold will identify more heteroresistance than those using a 5% threshold, even with identical samples.^9^

### Comparative evaluation of screening methods

No single screening method is perfect; each possesses a unique profile of strengths and weaknesses that must be considered in the context of its intended use. Table 1 provides a comparative summary of the key methods discussed.

**Table 1:** Comparison of major screening methods for TB heteroresistance detection

Specific Method(s)	Principle	Typical LOD^a^ for Heteroresistance	Turnaround Time	Key Advantages	Key Limitations
MGIT 960, APM	bacterial growth in drug-containing media	∼1%^7^^,^^10^	2–8 weeks	detects all resistance mechanisms; established gold standard.	extremely slow; requires BSL-3 containment; labor-intensive.
ODELAM	single-cell growth dynamics via microscopy	∼0.1%^33^	30–48 h	rapid; high sensitivity; mechanism-independent.	requires specialized equipment; lower throughput.
Xpert MTB/RIF (Ultra)	real-time PCR	10%–70%^10^^,^^37^	<2 h	extremely rapid; automated; widely deployed for POC use.	very low sensitivity for heteroresistance; limited gene targets.
Line Probe Assay (LPA)	reverse hybridization of PCR products	5%–10%^26^^,^^37^	1–2 days	relatively fast; detects multiple mutations simultaneously.	moderate sensitivity; can miss novel mutations.
Sanger Sequencing	chain-termination DNA sequencing	∼15%–25%^9^^,^^19^	1–3 days	provides exact sequence; confirms mutation identity.	low sensitivity for mixed populations; not quantitative.
WGS/TDS	massively parallel sequencing	∼1%–5% (depth-dependent)^42^^,^^57^	3–7 days	comprehensive genomic data; quantifies variants; discovers novel mutations.	costly; complex bioinformatics; requires high DNA input or enrichment.
ddPCR	absolute quantification via sample partitioning	∼0.1%–0.5%^50^^,^^52^	<1 day	highest sensitivity and precision for quantification; rapid.	targets known mutations only; lower multiplexing capability.

MGIT, growth indicator tube; APM, Agar proportion method; ddPCR, droplet digital PCR; LOD, limit of detection; TDS, targeted deep sequencing; WGS, whole genome sequencing.

a LOD values represent the minimum frequency of resistant subpopulation detectable under optimal conditions. Actual performance may vary based on sample quality, bacterial load, and specific mutations.

In summary, a clear trade-off exists between speed and sensitivity. Rapid point-of-care tests like Xpert offer unparalleled speed but are the least sensitive for detecting heteroresistance. LPAs and Sanger sequencing represent an intermediate group, while phenotypic methods, though slow, retain high sensitivity. The most powerful tools for investigating and quantifying heteroresistance are undoubtedly NGS and ddPCR, which combine high sensitivity with the ability to precisely measure the proportion of resistant subpopulations. The selection of an appropriate diagnostic strategy, therefore, depends on balancing the clinical need for rapid results against the risk of missing low-frequency but potentially significant resistant subpopulations. This diagnostic dilemma directly informs the clinical consequences of heteroresistance, which will be explored in the following section.

## Impact of TB heteroresistance on treatment outcomes and prognosis

The clinical relevance of TB heteroresistance is ultimately determined by its impact on patient outcomes. The administration of a standard, seemingly appropriate, treatment regimen inadvertently selects for the pre-existing resistant subpopulation, leading to a cascade of negative consequences including diminished treatment efficacy, increased rates of failure and recurrence, and overall a poorer long-term prognosis for the patient.

### The effect on treatment efficacy and failure

Kargarpour Kamakoli et al. found heteroresistance in 75% of treatment failures versus 25% of treatment successes (crude OR = 9.0, 95% CI: 3.2–25.3, *p* < 0.001). After adjusting for baseline resistance profile, HIV status, and previous treatment, the association remained significant (aOR = 7.3, 95% CI: 2.4–22.1)^5^. Similarly, Shin et al. reported an adjusted odds ratio of 5.42 (95% CI: 1.68–17.5) for unfavorable outcomes in mixed infections, controlling for age, sex, HIV status, baseline drug resistance, and treatment regimen.^6^ The clinical observation is clear: the presence of heteroresistance undermines the very foundation of standardized therapy. For example, a patient with undetected RIF heteroresistance who is placed on a standard first-line regimen is effectively receiving INH monotherapy for the resistant subpopulation, a known recipe for the amplification of resistance and therapeutic failure. The evolutionary dynamics of this process within a single patient have been elegantly chronicled in longitudinal studies. The case documented by Hjort et al., tracking a patient over nine years, provides a vivid illustration of this stepwise evolution. The infecting strain initially displayed heteroresistance to RIF, with multiple *rpoB* mutations coexisting. Over time and under continuous, albeit fluctuating, drug pressure, a clonal sweep occurred, leading to the fixation of the most fit resistant variant and the emergence of XDR-TB.^58^ Another compelling longitudinal case study by Sonnenkalb et al. tracked a patient for 27 years, showing how suboptimal treatment regimens containing too few effective drugs fueled a competitive “evolutionary arms race” among heteroresistant subpopulations, ultimately resulting in a pan-resistant infection.^59^

This mechanism also helps explain cases of apparent “relapse” where the recurring strain exhibits a more extensive resistance profile than the initial isolate. The standard regimen successfully cleared the susceptible population but allowed the MDR subpopulation to flourish, leading to a “relapse” with a different, more resistant genotype.^60^ These cases underscore that without the ability to detect the full spectrum of resistance at baseline, clinicians risk selecting for the most dangerous bacteria in their patients.

The relationship between assay sensitivity and clinical outcomes reveals a critical diagnostic-therapeutic gap. Studies using high-sensitivity methods (ddPCR, deep sequencing with >1000× coverage) that detect heteroresistance at 1%–5% frequency consistently report stronger associations with poor outcomes (pooled OR = 6.8, 95% CI: 3.9–11.8) compared to studies using conventional methods detecting at >10% frequency (pooled OR = 3.2, 95% CI: 1.8–5.7)^4^. This gradient effect suggests that low-frequency variants missed by routine diagnostics contribute substantially to treatment failure. For example, Metcalfe et al. found that among patients with discordant Xpert-negative/culture-positive rifampicin resistance, 73% had heteroresistance at 5%–20% frequency detectable only by deep sequencing. Conversely, patients with heteroresistance detected and treated appropriately showed outcomes comparable to those with homogeneous resistance (*p* = 0.31).^49^ This synthesis indicates that the “hidden” heteroresistance below current diagnostic thresholds represents a clinically actionable finding that, if detected, could prevent treatment failure in a significant proportion of patients.

### The unresolved question: What is the clinically significant threshold?

While the link between heteroresistance and poor outcomes is increasingly evident, a critical and unresolved question remains: what is the minimum frequency of a resistant subpopulation that is clinically significant? The traditional 1% threshold for phenotypic DST is historically rooted but not necessarily grounded in robust clinical outcome data for all drugs. The advent of ultra-sensitive technologies like deep sequencing and ddPCR, capable of detecting variants at frequencies of 0.1% or even lower, forces the clinical community to confront this question.^50^^,^^61^

Current evidence regarding the clinical significance of heteroresistance is derived from distinct yet inconclusive lines of investigation. *In vitro* models, such as hollow fiber system studies, demonstrate that suboptimal drug exposure can rapidly amplify minority resistant subpopulations from under 0.1%–0.2% to dominance within a 28-day treatment simulation.^62^ In contrast, analyses of clinical retrospective cohorts, Chen et al. analyzed 174 drug-susceptible TB patients retrospectively, finding no association between heteroresistance <1% (detected by deep sequencing) and treatment outcomes (*p* = 0.43).^63^ The available prospective clinical data, while limited to cohorts of several hundred patients with drug-resistant tuberculosis, similarly indicate no significant difference in outcomes for those harboring heteroresistance at frequencies between 1% and 5% (HR = 1.12, 95% CI: 0.78–1.61).^20^

This discrepancy highlights a complex interplay between the frequency of the resistant subpopulation, the specific drug and mutation in question (as different mutations cf. varying levels of resistance and fitness costs), the overall potency of the therapeutic regimen, and host immune factors. It is likely that there is no single universal threshold for clinical significance; rather, the risk is context-dependent. Establishing drug-specific and clinically validated thresholds for heteroresistance is a paramount research priority, essential for translating the data from highly sensitive diagnostics into meaningful clinical action and avoiding both under- and over-treatment.

## Preventive and control measures for TB heteroresistance

Addressing the challenge of tuberculosis heteroresistance demands a multi-pronged strategy that extends from public health interventions to clinical practice and laboratory diagnostics. The core principle underlying these measures is the prevention of the emergence and selection of resistant subpopulations. This involves strengthening fundamental TB control efforts, enhancing the precision and timeliness of diagnostics, optimizing therapeutic regimens, and ensuring rigorous oversight of anti-TB drug use.

### Strengthening tuberculosis prevention and control work

At the broadest level, preventing the rise of heteroresistance is intrinsically linked to the fundamental goals of TB control: interrupting transmission and ensuring high cure rates. Every successfully treated case of drug-susceptible TB prevents the potential for acquired resistance, including heteroresistance, to develop. This requires robust public health programs focused on active case finding, contact tracing, and ensuring treatment adherence through directly observed therapy (DOT) or other patient-centric support systems. Furthermore, molecular epidemiological studies that combine spatial analysis with genotyping, such as the work by Faye et al. in South Africa, are instrumental in identifying transmission hotspots of specific strains, including those associated with mixed infections or heteroresistance.^64^ By pinpointing these clusters, public health officials can direct resources more effectively to interrupt ongoing transmission chains and prevent the community-level spread of complex resistant populations.

### Improving the accuracy and timeliness of diagnosis

A critical leverage point for controlling heteroresistance is at the diagnostic stage. As previously discussed, standard molecular tests like Xpert may lack the sensitivity to detect low-frequency resistant variants, leading to the misclassification of patients and the prescription of ineffective regimens.^10^ The path forward involves a strategic integration of more sensitive technologies into diagnostic algorithms.

First, there is a compelling need to develop and deploy rapid diagnostic tests with a lower limit of detection for heteroresistance. Technologies like ddPCR and TDS have demonstrated the ability to detect subpopulations well below the 1% threshold.^42^^,^^50^ Jouet et al. and Chen et al. have shown the feasibility of using TDS directly on sputum samples, which could drastically reduce diagnostic delays and provide a comprehensive resistance profile, including quantitative information on heteroresistant variants, at the outset of treatment.^43^^,^^45^

Second, for cases with discordant results between genotypic and phenotypic tests, a structured investigation protocol, as proposed by Hofmann-Thiel et al., should be implemented.^11^ This includes repeating tests, confirming sample identity, and ultimately using a high-resolution method like WGS to resolve the discrepancy. In settings where advanced diagnostics are unavailable, retaining capacity for high-quality pDST remains crucial, as it provides a safety net for detecting resistance missed by less sensitive molecular assays.^7^

It is worth noting that, while ddPCR and deep sequencing offer superior sensitivity for heteroresistance detection, their implementation in resource-limited settings faces substantial barriers. Digital PCR requires specialized equipment costing $50,000–100,000, with per-sample costs of $20–50 compared to $10 for Xpert. Technical expertise for operation and maintenance is limited in many high-burden countries. Deep sequencing faces even greater challenges: equipment costs exceed $100,000, bioinformatics infrastructure is essential, and turnaround times of 3–7 days exceed the WHO’s 48-h target for rapid DST. However, emerging solutions show promise: portable sequencing devices (Oxford Nanopore MinION, ∼$1,000) have been successfully deployed in Uganda and Madagascar for targeted sequencing, with costs approaching $30 per sample. Centralized reference laboratory networks, as implemented in South Africa’s NGS surveillance program, offer a hybrid model where samples are transported for advanced testing while maintaining point-of-care Xpert for initial screening. Cost-effectiveness analyses suggest that detecting heteroresistance to prevent MDR-TB amplification may justify higher upfront diagnostic costs in specific scenarios.^65^

### Optimizing treatment regimens

The therapeutic management of TB, particularly when heteroresistance is known or suspected, must be guided by the principle of using a sufficient number of effective drugs to suppress all bacterial subpopulations. The study by Chen et al. provided striking evidence that treatment failure in MDR-TB was almost exclusively seen in patients receiving fewer than four effective drugs, powerfully illustrating the need for regimens robust enough to overcome pre-existing resistance.^13^

When heteroresistance to a key drug like rifampicin is detected, the patient should be managed as if they have MDR-TB, and the regimen should be adjusted accordingly by adding effective second-line agents. For newly developed drugs like bedaquiline, where heteroresistance is already a documented concern, early and accurate resistance testing is paramount to preserve their efficacy.^21^^,^^66^ Moreover, pharmacokinetic and pharmacodynamic (PK/PD) principles must be applied to optimize dosing. PK/PD optimization strategies must consider drug-specific and mutation-specific factors. For rifampicin, achieving Cmax/MIC >175 is associated with suppression of resistance amplification. Standard dosing (10 mg/kg) achieves this target for wild-type bacteria (MIC = 0.125 μg/mL) but fails for low-level resistant mutants like S531L (MIC = 2–4 μg/mL). High-dose rifampicin (35 mg/kg) can achieve suppressive concentrations even for 10% heteroresistant populations harboring S531L mutations.^67^ For fluoroquinolones, the mutant prevention concentration (MPC) for gyrA mutations is 4- to 8-fold higher than for wild-type. Standard levofloxacin dosing (750 mg) achieves AUC/MIC >100 for wild-type but fails to suppress heteroresistant populations with D94G mutations even at 1% frequency. Dose escalation to 1000–1250 mg or addition of adjuvants may be required.^68^ This suggests that for certain drugs, achieving high bactericidal concentrations at the site of infection are critical to ensuring the elimination of less-susceptible subpopulations. The concept of “lethality enhancement,” for example by using adjuncts like N-acetyl cysteine to increase the killing power of fluoroquinolones, represents another innovative therapeutic avenue to combat both tolerance and heteroresistance.^69^

### Strengthening drug management and supervision

Finally, programmatic and policy-level interventions are essential. Current WHO guidelines^70^ recommend that any detection of rifampicin resistance, regardless of the proportion of resistant bacteria, should prompt initiation of an MDR-TB regimen. This recommendation is based on evidence that even low-level rifampicin resistance is associated with poor outcomes on first-line therapy. The WHO explicitly states: “Patients with rifampicin-resistant TB (with or without isoniazid resistance) should be treated with an MDR-TB regimen.” National guidelines from high-burden countries align with this approach; for example, South African guidelines (2023) specify that any rifampicin resistance detected by Xpert or LPA warrants MDR-TB treatment, pending confirmatory testing. Every missed dose provides an opportunity for bacterial regrowth and the potential selection of resistant mutants. By ensuring patients complete their full course of effective therapy, the risk of amplifying a minor heteroresistant population into a full-blown resistant infection is significantly mitigated. This holistic approach, combining public health vigilance, diagnostic innovation, therapeutic optimization, and strict drug management, is essential to contain the threat posed by tuberculosis heteroresistance.

### Pragmatic framework for managing detected heteroresistance

While awaiting formal guidelines, we propose the following evidence-informed framework for managing detected heteroresistance:

*For Rifampicin*: (1) any detection (≥1%): treat as MDR-TB per WHO recommendations; (2) consider high-dose rifampicin (30–35 mg/kg) if heteroresistance is 1%–10% and no other first-line resistance.

*For Isoniazid:* (1) 10% heteroresistance: exclude from regimen; (2) 5%–10%: consider high-dose isoniazid (10–15 mg/kg) with close monitoring; (3) <5%: standard dose with enhanced adherence support; *For Fluoroquinolones:* (1) 5% heteroresistance: use alternative drug or higher dose (levofloxacin 1000–1250 mg); (2) 1%–5%: Consider therapeutic drug monitoring to ensure adequate exposure; (3) <1%: standard dosing with monthly culture monitoring.

*For Bedaquiline/Linezolid:* any detection: given limited alternatives, avoid if possible; if essential, combine with ≥3 other effective drugs.

*General principles:* (1) increase frequency of culture and DST monitoring (monthly for first 3 months); (2) ensure ≥4 effective drugs in regimen when heteroresistance detected; (3) consider therapeutic drug monitoring for key drugs; (4) document and report to surveillance systems.

This framework requires validation through prospective studies but provides a rational approach based on current evidence.

## Conclusion

This review has systematically charted the landscape of tuberculosis heteroresistance, illuminating it as a critical and pervasive challenge that complicates nearly every aspect of TB control. The coexistence of drug-susceptible and drug-resistant bacterial subpopulations within a single host is not a rare anomaly but a common evolutionary waypoint on the path to fixed resistance. Its documented prevalence, which varies significantly depending on the drug and diagnostic modality, underscores a global problem that has been linked unequivocally to adverse clinical outcomes, including diminished therapeutic efficacy, treatment failure, and the emergence of more formidable drug-resistant strains. Looking forward, addressing the multifaceted threat of heteroresistance requires a concerted and strategic research effort focused on several key fronts. Paramount among these is the need to define clinically significant thresholds for heteroresistance. While technology now allows for the detection of resistant variants at frequencies below 0.1%, the clinical implication of these minor populations remains undefined.

Secondly, the standardization of advanced diagnostic platforms is imperative. As highlighted by the variability in WGS performance across different laboratories, there is an urgent need for consensus on best practices, including minimum standards for sequencing depth, validated bioinformatics pipelines for variant calling, and the curation of comprehensive and regularly updated resistance mutation catalogs. Such standardization is essential to ensure that results are accurate, reproducible, and comparable, thereby fostering confidence in their clinical utility.

Furthermore, a deeper investigation into the fundamental biology of heteroresistance is warranted. Research should explore the host and pathogen factors that govern the emergence and maintenance. Questions regarding the role of spatial compartmentalization within lung lesions in sheltering diverse subpopulations, the fitness costs and benefits associated with specific resistance mutations, and the impact of the host immune response in containing or selecting for these variants remain largely unanswered. Answering them will provide a more complete picture of within-host dynamics and may reveal novel therapeutic targets.

The ultimate goal is to translate this knowledge into improved clinical practice. This involves not only the development of novel therapeutic strategies—such as PK/PD-optimized dosing regimens or ajuvant therapies that enhance drug lethality—but also the thoughtful integration of sensitive diagnostics into clinical care pathways, particularly in high-burden, resource-limited settings. By bridging the gap between our growing understanding of heteroresistance and our clinical response, the global health community can take a significant step toward neutralizing this insidious driver of drug resistance and advancing the goal of a world free of tuberculosis.

Finally, this review presents suggestions for both short-term research and long-term policies. (1) Immediate priorities (0–2 years): implement reflex testing with more sensitive methods (LPA or targeted sequencing) for all Xpert-negative/culture-positive cases; establish national reference laboratories with ddPCR or deep sequencing capability for discordant DST results; update treatment guidelines to explicitly address management of detected heteroresistance. (2) Medium-term goals (2–5 years): develop and validate point-of-care tests with <5% heteroresistance detection limits; conduct prospective trials to establish clinically relevant heteroresistance thresholds for key drugs; standardize bioinformatics pipelines and create quality assurance programs for NGS-based resistance detection. (3) Long-term vision (5–10 years): integrate real-time genomic surveillance with clinical care to enable personalized TB therapy; develop novel therapeutics targeting persistence and heterogeneity mechanisms; establish global heteroresistance surveillance networks to track emergence patterns.

## Data and code availability

Not applicable.

## Acknowledgments

This work was supported by the Doctoral Research Initiation Fund (2024BKQ011) and the Research Development Fund Project of the Affiliated Hospital of Shandong Second Medical University (2025FYQ034).

## Author contributions

Literature search: X.D., K.S., H.Z., and Y.C.; literature screening and data extraction: X.D., K.S., and H.Z.; data interpretation and synthesis: X.D., K.S., and Y.C.; writing: X.D., H.Z., and Y.C. All authors read and approved the final version of the manuscript.

## Declaration of interests

The authors declare no competing interests.
